# Supplementary figures and images for: Artemether Attenuates Gut Barrier Dysfunction and Intestinal Flora Imbalance in High-Fat and High-Fructose Diet-Fed Mice
Source: Nutrients. 2023 Nov 21;15(23):4860. doi: 10.3390/nu15234860 (PMC10707945; doi:10.3390/nu15234860)

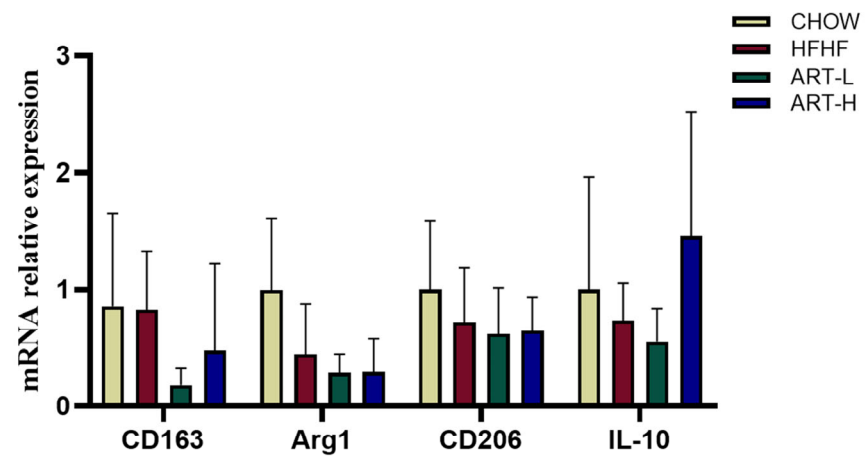

**Figure S1.** Effects of artemether intervention on M2 type transformation of macrophages.

Supplement: Supplementary file 1 [file nutrients-15-04860-s001.zip › nutrients-2691608-supplementary.pdf]
